# Pisa Syndrome in Parkinson's Disease Is Associated With Specific Cognitive Alterations

**DOI:** 10.3389/fneur.2019.00577

**Published:** 2019-05-31

**Authors:** Carlo Alberto Artusi, Elisa Montanaro, Sara Tuttobene, Alberto Romagnolo, Maurizio Zibetti, Leonardo Lopiano

**Affiliations:** Department of Neuroscience “Rita Levi Montalcini”, University of Torino, Turin, Italy

**Keywords:** Pisa syndrome, Parkinson's disease, cognition, neuropsychology, self-perception

## Abstract

**Background:** Pisa syndrome (PS) is a lateral flexion of the trunk frequently associated with Parkinson's disease (PD). The pathophysiology of PS remains unclear, but the role of cognitive deficits has been postulated.

**Methods:** We included 12 consecutive PD patients with PS (PS+) and 12 PD patients without PS (PS–) matched for gender, age, level of education, PD duration, and PD stage. As primary aim, we compared the neuropsychological scores of 16 tests evaluating 6 cognitive domains between PS+ and PS–. Additionally, we evaluated the presence of misperception of the trunk position in PS+, defined as a mismatch between the objective vs. subjective evaluation of the trunk bending angle >5°, and analyzed whether a correlation exists between the misperception of the trunk position and alterations in the visual-spatial abilities.

**Results:** PS+ group showed significantly worse performances in the visual-spatial abilities (*p*: 0.008), attentional domain (*p*: 0.001), and language domain (*p*: 0.023). No differences were found in the other cognitive domains nor in the general cognitive assessment. All PS+ patients showed a misperception of the trunk position, with an average underestimation of the trunk bending angle of 11.7° ± 4.3. The degree of misperception of the trunk position showed a trend toward a correlation with the visual-spatial scores (*p*: 0.089).

**Conclusions:** The study reveals an association between PS and specific cognitive alterations, suggesting a possible link between the abnormal posture of PD patients with PS and their cognitive functions.

## Introduction

Pisa syndrome (PS) is a posture abnormality characterized by lateral flexion of the trunk appearing or worsening while standing or walking and improving with passive mobilization and supine positioning (1). PS has an estimated prevalence of 7.4–10.3% in Parkinson's disease (PD), and demonstrated a relevant impact on patients' disability, being associated with low-back pain, imbalance, and quality of life impairment (1, 2). The pathophysiology of PS is still unclear, and different hypotheses have been postulated over time, which can be summarized in two main groups (1): a central hypothesis, encompassing a hyperactivation of axial muscles related to an imbalance of basal ganglia network output or an altered sensory-motor integration; and a peripheral hypothesis, related to musculoskeletal pathology with myopathic alterations in paraspinal muscles (2). Recent evidence, however, suggests that cognitive processes are involved in the pathophysiological mechanisms of PS (3). In particular, preliminary data showed that verticality perception deficits and altered visual-spatial functions might represent a typical feature of PD patients with PS (3, 4).

In this pilot cross-sectional study, we sought to analyze the differences in the cognitive profile of PD patients with and without PS. As primary aim, we compared the neuropsychological scores of 6 cognitive domains between PD patients with PS and a control group of matched PD patients without posture alterations. As secondary aims, we evaluated the presence of misperception of the trunk position in PD patients with PS, and analyzed whether a correlation exists between the misperception of the trunk position and alterations in the visual-spatial abilities.

## Materials and Methods

### Study Population

In this pilot cross-sectional study we included all consecutive consenting patients attending the Movement Disorder Center of the Turin University Hospital between January 2018 and June 2018 satisfying the following inclusion criteria: diagnosis of idiopathic PD (5) and presence of PS, defined as minimum 10° lateral flexion of the trunk that could be reduced by passive mobilization or supine positioning (PS+ group) (1). We excluded patients with: (a) age >80 years, (b) orthopedic issues complicating PS, encompassing vertebral fractures, severe osteoporosis, spondylodiscitis, idiopathic scoliosis with vertebral rotation, and history of major spine surgery, and (c) patients treated with cholinesterase inhibitors, neuroleptics, tricyclic antidepressants, and deep brain stimulation.

Controls (PS– group) were PD patients without PS or other posture alterations matched in a case-control design for the following clinical and demographic features: gender, age (±3 years), level of education, PD duration (±3 years) and stage of PD, according to the Hoehn and Yahr (HY) score (6). The same exclusion criteria applied for PS+ patients were used for PS– patients.

### Outcome Measures

All patients underwent an extensive clinical, neurological and neuropsychological assessment performed in their best clinical condition, defined as the time-frame in which the patient experiences the best control of PD symptoms, according to the judgment of the patient himself and the investigator.

#### Neuropsychological Assessment

Patients underwent a brief cognitive screening instrument, the Montreal-Cognitive Assessment (MoCA) (7). Then they were submitted to an extensive neuropsychological battery, assessing six cognitive domains: visual-spatial, memory, reasoning, attention, executive functions, and language.

Visuo-spatial abilities were assessed by Benton Judgment of Line Orientation (BJLOT) (8), Constructional Apraxia Test (CAT) (9) and Rey–Osterrieth Complex Figure Test (copy—ROCFT) (10). The memory domain was assessed by Italian Bi-syllabic Words Repetition Test (BWRT) (9), Digit Span (DST) (11), and Corsi's Block Tapping Test (CBT) (9) for verbal, numerical and spatial short-term memory; and by Rey auditory verbal learning (RAVLT) (12) and Rey–Osterrieth Complex Figure Test (delayed recall—ROCFT) for learning (10). Non-verbal reasoning was assessed by Raven Color Progressive Matrices Test (RCPMT) (13). The attentional domain was assessed by Digit Cancellation Test (DCT) (9) and Trail Making Test A (TMT A) (14). Executive functions were assessed by Trail Making Test B (TMT B) (14), TMT B-A (14), Frontal Assessment Battery (FAB) (15), and Modified Card Sorting Test (MCST) (16). The language was assessed by Phonemic Verbal Fluency (PVF) (9) and Semantic Verbal Fluency (SVF) tasks (17).

Age and education appropriate normative data were used to adjust the raw scores obtained by patients in each neuropsychological test. To obtain comparable categorical data the adjusted score of each test was rated as: 0, normal performance; 1, limited performance; 2, moderate impairment; 3, severe impairment, according to the equivalent scores based on the percentiles of population normative data (18–20). Then, for each cognitive domain, we obtained a Cognitive Index (CI), corresponding to the average value (0–3) of the related tests (18).

#### Misperception of the Trunk Position

The misperception of the trunk position was calculated as the mismatch between the objective vs. subjective evaluation of the trunk bending angle. In absence of validated criteria for the clinically meaningful change in trunk alterations, we relied on a cut-off of 5° as previously reported (21), and considered a mismatch >5° as a misperception of the trunk position.

**Figure 1:**
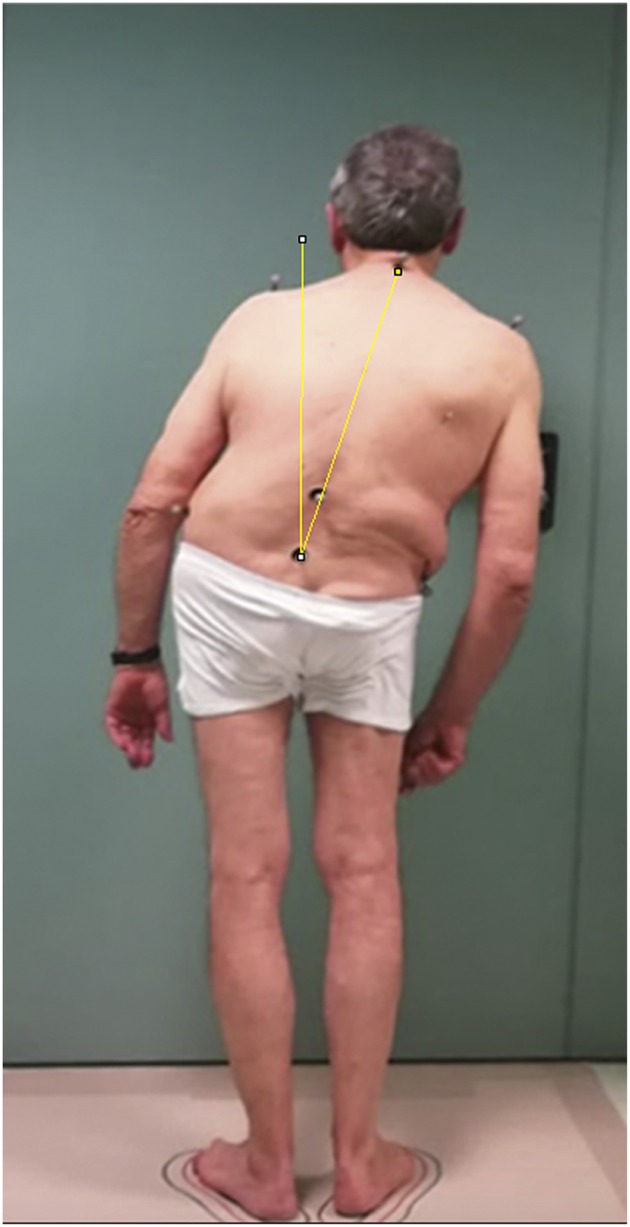
Example of the objective measurement of lateral trunk flexion in a patient with Pisa syndrome. The objective trunk position was calculated using “ImageJ” software, an open source image processing program developed by the National Institute of Health and validated for posture analysis (22). The patient gave his written informed consent for the publication of the picture.

The objective trunk position was calculated using “ImageJ” free software by capturing planar view photos of the patient standing in front of a wall goniometer, drawing a line between the 7th spinous process and the midpoint of the superior posterior iliac spine, and calculating the angle between this line and the vertical passing through the midpoint of the superior posterior iliac spine (Figure 1) (22).

The subjective trunk position was calculated by asking the patient to estimate his lateral trunk flexion angle specifying his/her own perceived trunk bending side and degrees while maintaining the standing position with open eyes (23).

Finally, further evaluation of an altered perception of the trunk position was obtained passively moving the patient with closed eyes from his natural trunk position to the vertical line (0°), and asking him to estimate if he still feels his trunk bending and, if so, on which side.

#### Demographic and Clinical Data

Other demographic and clinical data analyzed were: age, sex, age at PD onset, age at PS onset, PS duration, side of trunk deviation, MDS-UPDRS part III total score (6), HY score (6), pharmacological therapy, and Levodopa Equivalent Daily Dose (LEDD).

The following behavioral measures were also collected: depression, evaluated by means of the Beck Depression Inventory (BDI) (24); apathy, evaluated by means of the Apathy Scale (AS) (25); and anxiety, evaluated by means of the State-Trait Anxiety Inventory (STAI - X Form) (26).

### Statistical Analysis

Demographic and clinical features were summarized as mean ± standard deviation or percentages, as appropriate. The non-parametric Mann-Whitney test or the Fisher exact test was used to compare PS+ vs. PS– group neuropsychological and clinical data.

The correlation between the visual-spatial domain score and the degree of misperception of trunk position was performed by the Spearman correlation test.

All *p*-values reported are two-tailed and a *p* < 0.05 was considered statistically significant. Data were analyzed using the Statistical Package for the Social Sciences (SPSS 22 for Mac, Chicago, IL).

The local ethical committee approved the study and all patients included gave their written informed consent.

## Results

A total of 24 PD patients were enrolled in the study according to the inclusion/exclusion criteria: 12 patients for the PS+ group and 12 patients for the PS– group. The demographic and clinical features of patients are summarized in Table 1. No significant difference was found for age, education, PD duration, PD phenotype, HY stage, and LEDD between the 2 groups (Table 1). The only difference in the demographic and clinical features analyzed was in the MDS-UPDRS III score, with PS+ patients showing a higher score (49.3 ± 10.4 vs. 29.3 ± 12.5; *p*: 0.002).

**Table 1:** Patients' demographic and clinical features.

	**Overall sample**	**PS+ group**	**PS– group**	***p*-value**
Sex (male/female)	16/8	8/4	8/4	1.000
Age (years)	67.6 ± 9.9 (48–80)	67.8 ± 9.9 (49–80)	67.3 ± 10.3 (48–80)	0.839
Education (years)	10.5 ± 3.4 (5–15)	10.6 ± 3.5 (5–15)	10.3 ± 3.6 (5–14)	0.929
Disease duration (years)	9.5 ± 4.7 (3–17)	10.4 ± 4.9 (3–17)	8.5 ± 4.4 (3–17)	0.354
Age at diagnosis (years)	58.2 ± 10.2 (38–73)	57.5 ± 10.4 (38–73)	58.8 ± 10.4 (40–73)	0.840
Age at PS onset (years)	63.2 ± 9.3 (47–77)	63.2 ± 9.3 (47–77)	n.a.	n.a.
PS duration (years)	3.2 ± 2.4 (0–7)	3.2 ± 2.4 (0–7)	n.a.	n.a.
Side of trunk deviation (right/left)	7/5	7/5	n.a.	n.a.
MDS-UPDRS III	39.3 ± 15.2 (13–67)	49.3 ± 10.4 (34–67)	29.3 ± 12.5 (13–51)	**0.002**
Hoehn & Yahr	2.5 ± 0.6 (2–4)	2.7 ± 0.5 (2–4)	2.2 ± 0.6 (2–4)	0.068
Phenotype (Tremor/Akinetic-rigid/Mixed)	0/18/6	0/9/3	0/9/3	0.104
LEDD (mg)	828.3 ± 316 (205–1400)	797.1 ± 303.4 (300–1350)	859.5 ± 341.5 (205–1400)	0.545
MMSE	27.9 ± 1.9 (23–30)	27.3 ± 2.1 (23–29)	28.5 ± 1.3 (26–30)	0.175
BDI II	11.7 ± 6.8 (0–24)	12 ± 7 (0–21)	11.5 ± 7 (5–24)	0.788
AS	14.4 ± 7.2 (3–28)	16.2 ± 7.5 (4–28)	12.9 ± 7 (3–26)	0.328
STAI X1	46 ± 11.7 (21–67)	47 ± 13.1 (21–60)	45.2 ± 11.4 (35–67)	0.563
STAI X2	42.3 ± 11.8 (22–60)	46 ± 12.6 (22–59)	39.4 ± 10.6 (26–60)	0.248

*All data are reported as mean ± standard deviation (range), with the exception of sex and phenotype. MDS-UPDRS, Movement Disorders Society–Unified Parkinson's Disease Rating Scale; n.a., not applicable; LEDD, Levodopa Equivalent Daily Dosage; MMSE, Mini Mental State Examination; BDI II, Beck Depression Inventory II; AS, Apathy Scale; STAI X1, State-Trait Anxiety Inventory X Form (state anxiety); STAI X2, State-Trait Anxiety Inventory X Form (trait anxiety). Bold values mean statistically significant difference*.

### Neuropsychological Assessment

The global cognitive evaluation did not show a significant difference between the two groups, with a MOCA score of 21.7 ± 4.8 for PS+ vs. 23.5 ± 3.9 for PS– (*p*: 0.223).

**Figure 2:**
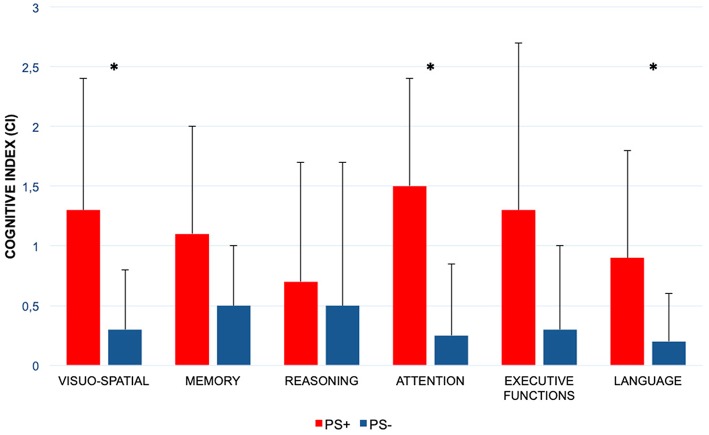
Comparison of the six cognitive domains scores between patients with Pisa syndrome and patients without posture alterations. PS+, patients with Parkinson disease and with Pisa syndrome; PS–, patients with Parkinson disease and without Pisa syndrome; ^*^statistically significant difference. Columns represent the mean values of the Cognitive Index, and bars indicate the standard deviation.

PS+ group showed a significantly worse performance in visual-spatial abilities (CI PS+ = 1.3 ± 1.1 vs. CI PS– = 0.3 ± 0.5; *p*: 0.008), attentional domain (CI PS+ = 1.5 ± 0.9 vs. CI PS– = 0.25 ± 0.6; *p*: 0.001), and language (CI PS+ = 0.9 ± 0.9 vs. CI PS– = 0.2 ± 0.4; *p*: 0.023). No significant differences were observed in the other cognitive domains evaluated (Figure 2; Table 2).

**Table 2:** Neuropsychological scores obtained by the overall sample and comparison between PS+ and PS– group.

	**Overall sample**	**PS+ group**	**PS– group**	***p*-value**
	**Mean ± standard deviation (range)**	**Mean ± standard deviation (range)**	**Mean ± standard deviation (range)**	
MoCA	22.6 ± 4.4 (13–30)	21.7 ± 4.8 (13–30)	23.5 ± 3.9 (15–27)	0.223
RCPMT	26.3 ± 7 (12–36)	25.7 ± 6.6 (16–35)	26.9 ± 7.6 (12–36)	0.664
BWRT	3.9 ± 0.9 (3–6)	3.6 ± 0.7 (3–5)	4.2 ± 1.1 (3–6)	0.102
DST	4.9 ± 1.3 (3–8)	4.8 ± 0.9 (4–6)	5.1 ± 1.6 (3–8)	0.721
CBT	4.3 ± 0.7 (3–6)	3.9 ± 0.5 (3–5)	4.7 ± 0.6 (4–6)	**0.007**
RAVLT–*Immediate recall*	35.1 ± 11.7 (15–59)	31.7 ± 12.6 (15–55)	38.6 ± 10.1 (24–59)	0.118
RAVLT–*Delayed recall*	6.9 ± 3.7 (0–13)	6.9 ± 3.7 (0–13)	7 ± 3.8 (2–13)	0.977
ROCFT–*Copy*	31 ± 5.6 (17–36)	27.9 ± 6.1 (17–36)	34.3 ± 2 (30–36)	**0.004**
ROCFT–*Delayed recall*	13.9 ± 6.4 (3–30)	11.9 ± 4.6 (4–18)	16.2 ± 7.5 (3–30)	0.056
DCT	42.6 ± 11.3 (15–60)	36.2 ± 10.6 (15–51)	49 ± 8.1 (36–60)	**0.003**
TMT A	63.6 ± 33.5 (22–143)	77.2 ± 35.2 (44–143)	49.9 ± 26.6 (22–114)	**0.040**
TMT B	188.2 ± 144.7 (42–532)	250.2 ± 158.6 (91–532)	126.2 ± 101.3 (42–400)	**0.018**
TMT B-A	130.1 ± 127.9 (20–400)	183.8 ± 145 (28–400)	76.3 ± 82.9 (20–319)	0.057
FAB	14.3 ± 2.9 (9–18)	13.2 ± 2.7 (9–17)	15.4 ± 2.7 (9–18)	0.050
MCST–*Categories*	4.4 ± 1.6 (2–6)	4.1 ± 1.7 (2–6)	4.7 ± 1.6 (2–6)	0.355
MCST–*Errors*	6.7 ± 5.3 (0–22)	6.2 ± 3.9 (0–11)	7.3 ± 6.6 (0–22)	0.766
MCST–*Perseverative errors*	3.6 ± 3.7 (0–11)	4.8 ± 4 (0–11)	2.4 ± 3.1 (0–10)	0.096
CAT	12 ± 1.8 (7–14)	11 ± 1.8 (7–13)	13.1 ± 1 (11–14)	**0.003**
BJLOT	21.7 ± 4.5 (13–29)	19.3 ± 4.4 (13–28)	23.4 ± 3.9 (17–29)	**0.039**
PVF	32.1 ± 14.6 (11–64)	27.2 ± 9.5 (11–40)	37.1 ± 17.4 (17–64)	0.204
SVF	20.8 ± 6.9 (12–36)	18.3 ± 6.5 (12–32)	23.4 ± 6.5 (12–36)	0.057

*MoCA, Montreal Cognitive Assessment; RCPMT, Raven Colored Progressive Matrices Test; BWRT, Italian Bi-syllabic Words Repetition Test; DST, Digit Span Test; CBT, Corsi's Block Tapping Test; RAVLT, Rey Auditory Verbal Learning test; ROCFT, Rey-Osterrieth Complex Figure Test; DCT, Digit Cancellation Test; TMT A, Trail Making Test A; TMT B, Trail Making Test B; TMT B-A, Trail Making Test B-A; FAB, Frontal Assessment Battery; MCST, Modified Card Sorting Test; CAT, Constructional Apraxia Test; BJLOT, Benton Judgment of Line Orientation Test; PVF, Phonological Verbal Fluency; SVF, Semantic Verbal Fluency. Bold values mean statistically significant difference*.

### Misperception of the Trunk Position

We found a misperception of the trunk position in 100% of PS patients (*n* = 12/12), with an average underestimation of the trunk bending angle of 11.7° ± 4.3. Moreover, all PS+ patients referred a subjective feeling of bending on the opposite side compared to their PS bending side when passively positioned on the vertical line (0°) with their eyes closed (e.g., patients with right-sided PS referred to bend on the left side and vice versa).

The degree of misperception of the trunk position showed a trend toward a correlation with the visual-spatial CI score (*p*: 0.089).

## Discussion

This pilot study supports the hypothesis of a specific neuropsychological profile of PD patients with PS. In fact, we found a worse performance in the visual-spatial, attentional, and language domains in PD patients with PS compared with a matched group of PD patients without posture alterations. Moreover, we found a misperception of the trunk position in all PS patients, which showed a possible correlation with the score of visual-spatial abilities.

The role of the Central Nervous System in the pathophysiology of PD-associated PS is sustained by a vast body of evidence (1); however, the exact pathophysiological mechanism underlying PS is still a matter of debate. Altered perception of verticality in PS patients has been reported by different studies (4, 27), and this defect is supposed to be associated with the impaired integration of visual, vestibular and somatosensory information. A recent study on 54 patients with PS demonstrated that vertical misperception is not only a feature of PS patients but also a relevant risk factor for PS development (4). To date, only one study investigated by means of a comprehensive neuropsychological battery the cognitive profile of patients with PS, concluding that PS may be associated with altered performance in attention and posterior cortical functions (3).

Our study confirms the presence of specific neuropsychological alterations in PS, endorsing a possible role for visual-spatial and attentional impairment. In particular, our data indicate a correlation between PS and specific cortical cognitive deficits, which can be strictly related to the perception of the trunk position in the space. Indeed, we also observed that all PS patients had a significant misperception of their trunk position, with a possible correlation between the severity of misperception and the severity of visual-spatial deficits. In addition, we found a worse performance in the language domain, particularly for the access to the semantic lexicon, an ability related to the infero-posterior temporal lobe function that involves the semantic representation of nouns or objects mostly constituted of perceptual/sensory content (28). Alterations in language performance in PD proved to be similar to those observed in Lewy Body dementia, typically characterized by visual-spatial deficits, and different to those observed in Alzheimer disease. This data, while requesting confirmation in larger studies, may suggest a further link between visuoperceptual dysfunctions and PS.

While some authors hypothesized the contribution of peripheral proprioceptive or vestibular apparatus alterations in the pathophysiology of PS, a recent study showed that most PS patients do not suffer from peripheral deficits (4), suggesting the involvement of higher subcortical and cortical networks. Moreover, PD patients with PS showed a higher tendency to have a veering gait compared to PD patients without PS (2), and this phenomenon has been correlated with visual-perceptual impairment (29). Accordingly, our results showed the presence of altered performance in neuropsychological tests related to visual-perceptual abilities. These findings represent a clue for the role of high cognitive functions in the pathophysiology of PS, highlighting the importance of early management of PS patients with both physical and neuropsychological rehabilitative programs (2).

The strength of our findings should be tempered by the small sample size, and the absence of a follow-up with the possibility to establish whether the cognitive deficits preceded or followed the onset of PS. Moreover, PS+ patients showed a higher MDS-UPDRS-III score than PS–. While this finding could be partly explained by the higher score obtained in the posture item by patients with PS, a more aggressive disease phenotype cannot be excluded in spite of the strict matching criteria applied. Finally, we did not investigate a possible vestibular dysfunction, which has been previously associated with impairment in visual-spatial abilities (30).

In conclusion, considering these limitations, our study reveals an association between PS and specific cognitive alterations, suggesting a potential contribution of cortical and subcortical dysfunctions in the pathophysiology of PS. A longitudinal, multi-center study is necessary to confirm these findings and to clarify the role of specific cognitive alterations as a risk factor for PS.

## Data Availability

Datasets are available on request. The raw data supporting the conclusions of this manuscript will be made available by the authors, without undue reservation, to any qualified researcher.

## Ethics Statement

This study was carried out in accordance with the recommendations of the institutional review board with written informed consent from all subjects. All subjects gave written informed consent in accordance with the Declaration of Helsinki. The protocol was approved by the Comitato Etico Interaziendale A.O.U. Città della Salute e della Scienza di Torino-A.O. Ordine Mauriziano-A.S.L. Città di Torino.

## Author Contributions

CA contributed to study concept and design, analysis and interpretation of data, and drafting of the manuscript. EM contributed to acquisition, analysis, and interpretation of data, and drafting of the manuscript. ST contributed to acquisition, analysis, and interpretation of data. AR contributed to statistical support, interpretation of data, and critical revision of the manuscript for intellectual content. MZ contributed to interpretation of data, critical revision of the manuscript for intellectual content. LL contributed to the study concept and design, critical revision of the manuscript for intellectual content, supervision of the study. All authors gave final approval of the version to be published.

## Contribution to the Field Statement

Pisa syndrome (PS) is a disabling posture alteration affecting almost 10% of patients with Parkinson's disease (PD). However, the pathophysiology of PS still needs to be elucidated, and the absence of known pathophysiology reflects on the absence of specific therapies. While proving the role of the central nervous system, some information provided by the literature seems to suggest a role of cognitive functions in determining PS. Nonetheless, to our knowledge, only one study investigated so far the cognitive profile of PS patients. The results of our study endorse the hypothesis of specific cognitive dysfunction associated with PS. Pointing out the role of visual-spatial abilities and attention deficits in PS patients, our findings provide an important piece of information in the debate on PS pathophysiology, at the same time highlighting the importance of early management of PS patients with both physical and neuropsychological rehabilitative programs.

## Disclosure

CA has received travel grants from Zambon and Abbvie. AR has received grant support and speaker honoraria from AbbVie, speaker honoraria from Chiesi Farmaceutici and travel grants from Medtronic, Lusofarmaco, and UCB Pharma. MZ has received speaker's honoraria from Medtronic, Lundbeck, UCB Pharma, and AbbVie. LL has received honoraria for lecturing and travel grants from, UCB Pharma, AbbVie, DOC, Zambon, and Bial. EM and ST reports no disclosures.

### Conflict of Interest Statement

The authors declare that the research was conducted in the absence of any commercial or financial relationships that could be construed as a potential conflict of interest.
